# Phase II Study of Platinum Re-administration in Non-small Cell Lung Cancer Following Chemoimmunotherapy Resistance

**DOI:** 10.7759/cureus.84709

**Published:** 2025-05-23

**Authors:** Kiichiro Ninomiya, Shinobu Hosokawa, Toshihide Yokoyama, Masaaki Inoue, Keisuke Sugimoto, Kenichiro Kudo, Toshiaki Okada, Tadashi Maeda, Haruyuki Kawai, Masayuki Yasugi, Koji Inoue, Shoichi Kuyama, Takashi Ninomiya, Isao Oze, Yoshinobu Maeda, Katsuyuki Hotta

**Affiliations:** 1 Center for Comprehensive Genomic Medicine, Okayama University Hospital, Okayama, JPN; 2 Respiratory Medicine, Japanese Red Cross Okayama Hospital, Okayama, JPN; 3 Respiratory Medicine, Kurashiki Central Hospital, Kurashiki, JPN; 4 Chest Surgery, Shimonoseki City Hospital, Shimonoseki, JPN; 5 Respiratory Medicine, Japanese Red Cross Kobe Hospital, Kobe, JPN; 6 Respiratory Medicine, National Hospital Organization Okayama Medical Center, Okayama, JPN; 7 Respiratory Medicine, National Hospital Organization Fukuyama Medical Center, Fukuyama, JPN; 8 Respiratory Medicine, National Hospital Organization Yamaguchi Ube Medical Center, Ube, JPN; 9 Internal Medicine, Okayama Saiseikai General Hospital, Okayama, JPN; 10 Respiratory Medicine, Chugoku Central Hospital, Fukuyama, JPN; 11 Respiratory Medicine, Ehime Prefectural Central Hospital, Matsuyama, JPN; 12 Respiratory Medicine, Iwakuni Medical Center, Iwakuni, JPN; 13 Respiratory Medicine, National Hospital Organization Shikoku Cancer Center, Matsuyama, JPN; 14 Division of Molecular Medicine, Aichi Cancer Center Research Institute, Nagoya, JPN; 15 Hematology, Oncology, and Respiratory Medicine, Okayama University Graduate School of Medicine, Dentistry, and Pharmaceutical Sciences, Okayama, JPN; 16 Center for Innovative Clinical Medicine, Okayama University Hospital, Okayama, JPN

**Keywords:** carboplatin, clinical trial, non-small cell lung cancer, platinum-free interval, re-administration

## Abstract

Platinum compounds represent standard treatments for various cancers. However, the efficacy of re-administrating platinum compounds has been demonstrated only in ovarian cancer and small cell lung cancer (SCLC), with its utility in non-small cell lung cancer (NSCLC) remaining unclear. Given that the prognosis of NSCLC has improved with effective treatments such as immune checkpoint inhibitors (ICIs), we propose a multicenter, single-arm, prospective phase II trial of re-administrating platinum-based combination therapy for NSCLC. Patients with NSCLC who have progressed after chemoimmunotherapy and exhibit a platinum-free interval of 85 days or more will receive carboplatin combination therapy. The drugs combined with carboplatin - pemetrexed, nab-paclitaxel, or gemcitabine - must not have been administered previously. The primary endpoint is progression-free survival (PFS), assessed using the Response Evaluation Criteria in Solid Tumors (RECIST) version 1.1. Assuming a threshold PFS of 4.2 months and an expected hazard ratio of 0.60, the required sample size is calculated as 40 patients to achieve 70% power with a 5% alpha error. Secondary endpoints included objective response rate, toxicity, and overall survival. This study will clarify the efficacy of re-administrating platinum compounds to patients with NSCLC who have developed resistance to chemoimmunotherapy.

## Introduction

Platinum compounds (cisplatin/carboplatin) constitute essential cytotoxic anticancer drugs and have served as standard treatments for non-small cell lung cancer (NSCLC) from the 1980s to the present day [1]. The treatment strategy for NSCLC has undergone a significant transformation with the introduction of molecularly targeted drugs and immune checkpoint inhibitors (ICIs). Nonetheless, platinum agents remain integral to first-line treatments, used in combination with molecularly targeted drugs [2] and ICIs [3]. The emergence of these new drugs has notably improved the prognosis [4]; however, challenges persist in selecting treatments for patients with NSCLC who develop resistance to these therapies, and the development of subsequent cytotoxic anticancer regimens remains insufficient.

The efficacy of re-administrating platinum-based chemotherapy has been established in other cancer types, such as ovarian cancer or small cell lung cancer (SCLC). For recurrent ovarian cancer, the effectiveness of re-administrating platinum drugs has been recognized in patients with a platinum-free interval (PFI) of six months or more [5], and combining platinum with other agents improves prognosis [6,7]. In SCLC, a phase III study showed that carboplatin plus etoposide significantly prolonged progression-free survival (PFS) compared to nogitecan monotherapy in patients with a PFI of three months or more [8]. For NSCLC, retrospective studies suggest greater benefit from platinum re-administrating in patients with longer PFIs [9,10]. However, no prospective clinical trials have evaluated the effectiveness of re-administrating platinum-based chemotherapy for NSCLC.

Based on this evidence, a single-arm trial was designed to establish new evidence for the re-administration of platinum agents for NSCLC. This study aims to clarify the efficacy of adding platinum agents to cytotoxic chemotherapy alone, which is the standard of care for second-line treatment.

Objective

This study will be conducted to evaluate whether the re-administration of a platinum agent enhances the efficacy of platinum-based chemotherapy and immunotherapy in patients with progressive disease.

## Materials and methods

Study design

**Figure 1:**
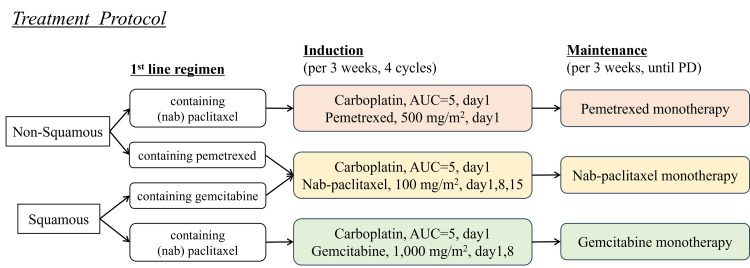
Treatment protocol AUC: area under the curve Credit: Image created by the authors

This study is structured as a multicenter, single-arm, prospective phase II trial. Figure 1 provides an overview of the study design. Written informed consent will be obtained from all patients prior to screening or inclusion procedures.

This study will be conducted in accordance with the principles of the Declaration of Helsinki, with the protocol approved by the institutional review board of each participating hospital.

Endpoints

The primary endpoint is PFS, assessed using the Response Evaluation Criteria in Solid Tumors (RECIST) version 1.1 [9]. Secondary endpoints include objective response rate, toxicity, and overall survival. Additionally, to identify biomarkers indicative of platinum compound efficacy, the correlation between efficacy and homologous recombination repair-related genes will be explored using results obtained from next-generation sequencing.

Eligibility criteria

Patients who had received platinum-based combination therapy with an ICI as initial treatment, with subsequent disease progression, and had a minimum interval of three months (85 days) since the last administration of a platinum agent (cisplatin or carboplatin) are enrolled. PFI was established based on phase 3 trials of SCLC that demonstrated efficacy for three months or more [8]. In cases with a driver gene mutation, targeted therapy has been administered, followed by disease progression. The main eligibility criteria are as follows: patients aged 20 years or older, an Eastern Cooperative Oncology Group (ECOG) performance status (PS) of 0 to 1, pathologically or cytologically confirmed lung cancer, and the presence of measurable lesions. Adequate organ function is also required, defined as follows: leukocytes ≥ 3,000/μL; absolute neutrophil count ≥ 1,500/μL; platelets > 100,000/μL; hemoglobin ≥ 9 g/dL; total bilirubin ≤ 1.5 mg/dL; aspartate aminotransferase/alanine transaminase levels < 100 IU/L; serum creatinine ≤ 1.5 mg/dL; and arterial oxygen partial pressure ≥ 60 mmHg or oxygen saturation by pulse oximetry ≥ 92% on room air. Exclusion criteria included a history of allergy to platinum-based drugs (cisplatin or carboplatin); active interstitial pneumonia on chest radiography; serious or uncontrollable complications; symptomatic brain metastases; a history of another cancer with a more defined prognosis than lung cancer and a disease-free period of less than three years; pleural or pericardial effusion requiring repeat drainage; and a requirement for urgent radiation therapy.

Statistical analysis

The primary objective of this study is to assess PFS following the re-administration of platinum-based combination therapy after chemoimmunotherapy. Based on a previous report, the threshold PFS for single-agent therapy as second-line treatment was 4.2 months [10], whereas the expected PFS is set at 7.0 months (hazard ratio: 0.60), deemed clinically significant. The required sample size, calculated using alpha error = 0.05 (one-sided) and power = 0.7, is 38 patients; accounting for potential ineligibility, the planned sample size for this phase II has been established as 40 patients.

Ethical considerations

This study will be conducted in accordance with the principles of the Declaration of Helsinki and the protocol approved by the Central Ethics Review Committee of Okayama University Hospital (CRB24-002). Written informed consent will be obtained from all patients prior to any screening or inclusion procedure. This protocol has been registered with the Japan Registry of Clinical Trials (jRCTs061240065).

Participating institutions

This study will be conducted by the Okayama Lung Cancer Study Group (OLCSG). The participating institutions include Okayama Red Cross Hospital, Kawasaki Medical School General Medical Center, Kurashiki Central Hospital, Shimonoseki City Hospital, Japanese Red Cross Kobe Hospital, National Hospital Organization Okayama Medical Center, National Hospital Organization Fukuyama Medical Center, National Hospital Organization Yamaguchi Ube Medical Center, Okayama Saiseikai General Hospital, Chugoku Central Hospital, Ehime Prefectural Central Hospital, National Hospital Organization Iwakuni Medical Center, National Hospital Organization Shikoku Cancer Center, and Okayama University Hospital. Each institution will obtain approval from the head of the facility to conduct the study after receiving approval from the central review board, prior to patient recruitment.

## Results

Planned interventions and assessments

Treatment Regimen

The treatment regimen will be selected based on the histological type and platinum-based combination therapy used in first-line treatment. For patients with squamous cell carcinoma, if paclitaxel or nab-paclitaxel has been previously administered, carboplatin plus gemcitabine will be chosen; if gemcitabine has been previously administered, carboplatin plus nab-paclitaxel will be selected. For patients with non-squamous cell carcinoma, if pemetrexed has been previously used, carboplatin plus nab-paclitaxel will be chosen; if paclitaxel or nab-paclitaxel has been previously used, carboplatin plus pemetrexed will be selected. Carboplatin will be administered at a dose calculated to produce an area under the concentration-time curve of 5 mg/mL^-1^/min for a maximum of four cycles every three weeks. Pemetrexed will be administered at 500 mg/m^2^ on day 1, gemcitabine at 1,000 mg/m^2^ on days 1 and 8, and nab-paclitaxel at 100 mg/m^2^ on days 1, 8, and 15. These drugs will be administered every three weeks in combination with carboplatin for the first four cycles, followed by continuation as single-agent maintenance therapy.

Follow-Up Schedule

All patients will be followed up for at least six months after registration. Toxicities will be graded using the National Cancer Institute Common Terminology Criteria for Adverse Events (CTCAE) version 5.0. Chest CT scans will be performed every six weeks from the start of treatment during the first four cycles and every nine weeks during maintenance therapy. Tumor assessments will be performed using the RECIST version 1.1. Overall survival is defined as the interval between the date of enrollment and the date of death or last follow-up visit. PFS is defined as the interval between the date of enrollment and the date of progressive disease or death.

## Discussion

Platinum-based chemotherapy is not currently recommended as second-line or later treatment for NSCLC in clinical practice guidelines [11,12]. However, its efficacy has been demonstrated in many retrospective observational studies. Miyawaki et al. reported that chemoradiotherapy followed by re-administrating platinum-based chemotherapy was more effective than single-agent therapy for disease progression after treatment in unresectable stage III NSCLC [13]. Murata et al. also reported that combination therapy with cisplatin, gemcitabine, and necitumumab was effective in cases previously treated with platinum-based chemotherapy and ICI [14]. Both reports suggest that longer PFI may prolong PFS in patients receiving platinum-based chemotherapy.

In NSCLC, treatment options following chemotherapy are limited, and there are no promising treatments other than docetaxel plus ramucirumab or other single-agent chemotherapy regimens [10,15,16]. Additionally, no prospective trials have demonstrated the efficacy of platinum-based regimens in the setting of prior platinum-based therapy. Therefore, the results of this trial may potentially provide new treatment options for clinical practice guidelines. However, further confirmatory trials are necessary to confirm these findings.

Given the high toxicity of platinum compounds, biomarkers predictive of their efficacy are needed. Homologous recombination repair deficiency, including *BRCA* gene mutations, may serve as a predictive factor for the efficacy of platinum compounds. In a case-control study of ovarian cancer, patients with *BRCA1* or* BRCA2* mutations showed higher response rates to platinum-based chemotherapy compared to patients without such mutations [17]. In preoperative chemotherapy for triple-negative breast cancer, homologous recombination deficiency not limited to *BRCA1/2* mutations showed high response rates to platinum-based chemotherapy [18]. As part of this study, comprehensive genomic profiling tests will be conducted to the extent feasible to explore the relationship between these genomic factors and efficacy for NSCLC.

This study has several limitations. First, this study is non-randomized, so the results are not conclusive and require further verification. In addition, enrollment is left to the discretion of the attending physician, which may introduce bias in patient background. This selection bias will be minimized by performing subgroup analyses based on histological type (squamous or non-squamous), PFI (six months or longer or shorter), and the effectiveness of initial chemoimmunotherapy.

## Conclusions

This study may be expected to reaffirm the significance of platinum compounds in NSCLC management, potentially establishing platinum-based combination therapy as a viable option for the second-line treatment of NSCLC.
